# Bruch’s Membrane: A Key Consideration with Complement-Based Therapies for Age-Related Macular Degeneration

**DOI:** 10.3390/jcm12082870

**Published:** 2023-04-14

**Authors:** Sarah Hammadi, Nikolaos Tzoumas, Mariantonia Ferrara, Ingrid Porpino Meschede, Katharina Lo, Claire Harris, Majlinda Lako, David H. Steel

**Affiliations:** 1 Biosciences Institute, Faculty of Medical Sciences, Newcastle University, Newcastle upon Tyne NE2 4HH, UK; 2 Sunderland Eye Infirmary, Queen Alexandra Rd., Sunderland SR2 9H, UK; 3 Manchester Royal Eye Hospital, Manchester M13 9WL, UK; 4 Gyroscope Therapeutics Limited, a Novartis Company, Rolling Stock Yard, 6th Floor, 188 York Way, London N7 9AS, UK; 5 Clinical and Translational Research Institute, Faculty of Medical Sciences, Newcastle University, Newcastle upon Tyne NE2 4HH, UK

**Keywords:** Bruch’s membrane, retinal pigment epithelium, choroid, age-related macular degeneration (AMD), complement system, complement therapies

## Abstract

The complement system is crucial for immune surveillance, providing the body’s first line of defence against pathogens. However, an imbalance in its regulators can lead to inappropriate overactivation, resulting in diseases such as age-related macular degeneration (AMD), a leading cause of irreversible blindness globally affecting around 200 million people. Complement activation in AMD is believed to begin in the choriocapillaris, but it also plays a critical role in the subretinal and retinal pigment epithelium (RPE) spaces. Bruch’s membrane (BrM) acts as a barrier between the retina/RPE and choroid, hindering complement protein diffusion. This impediment increases with age and AMD, leading to compartmentalisation of complement activation. In this review, we comprehensively examine the structure and function of BrM, including its age-related changes visible through in vivo imaging, and the consequences of complement dysfunction on AMD pathogenesis. We also explore the potential and limitations of various delivery routes (systemic, intravitreal, subretinal, and suprachoroidal) for safe and effective delivery of conventional and gene therapy-based complement inhibitors to treat AMD. Further research is needed to understand the diffusion of complement proteins across BrM and optimise therapeutic delivery to the retina.

## 1. Introduction

Age-related macular degeneration (AMD) is a leading cause of irreversible sight loss, affecting around 200 million globally, including 25% of those over 60 in Europe [1,2]. It leads to reduced quality of life, increased anxiety and depression, and has a substantial economic impact upwards of GBP 1.6 billion per year in the UK [3]. With ageing populations, its personal and socioeconomic impact will continue to grow. AMD involves progressive degeneration of the macula, including drusen formation and tissue atrophy, leading to severe central visual loss in its late stages [4]. While intravitreal anti-vascular endothelial growth factor (VEGF) therapy has been successful in improving outcomes in neovascular AMD, the majority of cases (95%) are non-exudative and, until recently, have been without any available treatment options. Moreover, the current treatments for neovascular AMD are inadequate as they do not halt the underlying degeneration, which ultimately results in atrophy or scarring in a significant number of cases, with around one-third of patients still experiencing visual decline despite treatment [5]. New therapies are needed to address this issue.

AMD, a multifactorial eye disease involving hypoxia, inflammation, vascular insufficiency, oxidative stress, and chronic immune activation, remains a complex and challenging research area [6]. The complement system, with its crucial role in immune and homeostatic functions, is a major contributor to these processes and a focus of current research for effective treatments [7]. AMD first manifests in the retinal pigment epithelium (RPE) and choroid. Bruch’s membrane, an elastin- and collagen-rich extracellular matrix situated between the retinal pigment epithelium and the choroid, has key structural and functional properties and acts as a molecular sieve to compartmentalise the retina from the systemic circulation. Systemic complement production, chiefly by hepatocytes, has well-known roles in homeostasis, but the effects of local complement production and action are less well recognised. Complement components are produced by several cell types within the retina. Müller cells produce most of the complement activators, retinal neurones factor I, while RPE cells express factor H and terminal complement components [8]. Thus, with the compartmentalisation afforded by BrM, this creates an environment discrete from many of the effects of systemic complement production. In vivo imaging of BrM and its surrounding area has seen significant progress in recent years, helping in early recognition of AMD. A detailed understanding of BrM properties is therefore crucial for both deciphering the pathogenesis of AMD and developing effective therapies.

This review covers the basics of BrM structure and function, its age-related changes visible on imaging, and its impact on complement dysfunction in AMD. We also discuss the potential and limitations of different delivery routes for effective and safe delivery of complement inhibition to the macula.

## 2. The Structure and Function of BrM

BrM is a 2–4 μm thin layer of extracellular matrix between the retina and choroid in the eye serving as a selectively permeable membrane between the RPE and choriocapillaris (CC). The RPE is a crucial monolayer of epithelial cells supporting the high metabolic activity of the neural retina through various key metabolic, transport, and immunoregulatory functions [9]. The CC, a 10 μm thick network of highly anastomosed and fenestrated capillaries, facilitates protein diffusion between the retina and choroid. The choroid, a vital vascular connective tissue, regulates blood flow and temperature and secretes growth factors to maintain eye homeostasis [10].

BrM is a continuous membrane posterior to the ora serrata, deficient only at the point of optic nerve insertion [11]. In 1971, Hogan described BrM as having five layers [12], which are described from inner to outer layers as follows:

- The RPE basement membrane is about 0.15 μm thick [13,14] and contains collagen IV α1–5, collagen V, heparan sulphate, chondroitin sulphate, laminins 1, 5, 10, and 11, and nindogen-1 [15,16,17,18,19,20].
- The inner collagenous layer (ICL) is about 1.4 μm thick containing crossed layers of collagen I, III, and V fibronectin, chondroitin sulphate, dermatan sulphate, apolipoprotein E (apoE), clusterin, heme, lipoproteins, and vitronectin [21,22,23,24,25,26].
- The central elastic layer (CEL) is about 0.8 μm thick, discontinuous in the macular region [27], and contains elastin fibres that are contiguous with the ICL and outer collagenous layer (OCL). The CEL is important for biomechanical properties, antiangiogenic barrier functions, and choroidal contractility [28].
- The OCL’s thickness ranges from 1 to 5 μm [29] and contains collagen I, III, and V, fibronectin, fibulin-5, chondroitin sulphate, lipoproteins, dermatan sulphate, clusterin, and apoE [17,19,24,26,30,31,32].
- The choroidal endothelial cell (CEC) basement membrane is about 0.07 μm thick and contains collagen IV, α1, α2, V, and VI, heparan sulphate, laminin, endostatin, and chondroitin sulphate [15,19,20,33,34]. It is discontinuous due to the presence of choroidal inter-capillary pillars between CC lumens [28].

**Figure 1:**
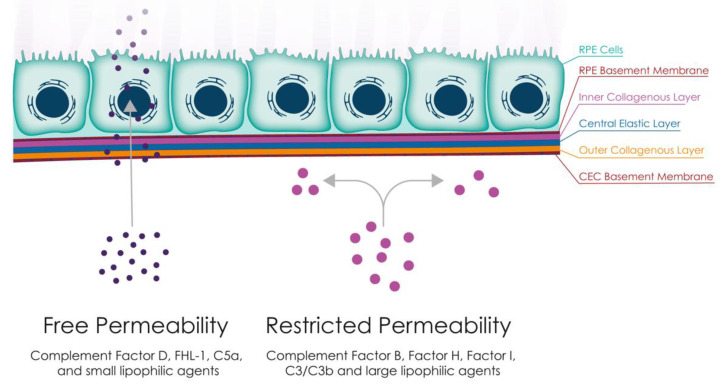
Complement diffusion through BrM. BrM has been shown to act as a barrier between the retina and choroid, allowing a limited number of complement proteins, such as FHL-1, factor D, and C5a, to diffuse through. This compartmentalisation may be exacerbated in ageing and disease, as lipid deposition in AMD has been shown to reduce FHL-1 diffusion. This could create two separate compartments for complement activation and regulation, namely the retinal and choroidal sides, with complement proteins remaining on their side of origin. Abbreviations: FHL-1, Factor H-like 1 protein; RPE, retinal pigment epithelium.

The exchange of nutrients, oxygen, minerals, and visual cycle by-products between the RPE and the CC is primarily regulated by BrM through passive diffusion. The permeability of BrM is largely determined by its structure and is mainly influenced by the molecular weight, size, and shape of the diffusing substance (Figure 1). While some complement proteins such as Factor H-like protein 1 (FHL-1), Factor D (FD), and C5a have been found to pass through BrM [35,36], it has been reported that C3a, despite its small molecular weight, is not able to do so. This observation may reflect the higher positive net charge of C3a at neutral pH [35], causing it to be trapped within the BrM, which is negatively charged due to the presence of glycosaminoglycans [35,37,38]. Post-translational modifications, such as N-linked glycosylation, may also impede diffusion of complement proteins such as Factor I (FI) through BrM, although these are not considered sufficient to explain the lack of diffusion of Factor H (FH) and Factor B (FB) [35,39]. Finally, solute transmission through BrM is determined by the hydrostatic pressure and the concentration gradient of molecules [14]. The CEL has the largest pore size and highest water diffusion [21], while the ICL has the smallest pore size and lowest water conductivity [21].

Additionally, BrM provides structural support to the RPE and has important transport functions. Its high elasticity, with an estimated modulus of 7 to 19 MPa [40], allows it to stretch and accommodate changes in intraocular pressure [28]. This is in stark contrast to the lower elasticity of the sclera, with a modulus of approximately 1.2 to 1.3 MPa [41], and the retina, which has an even lower modulus of 0.000208 MPa [42].

The BrM remodelling process involves three inactive forms of matrix metalloproteinases (MMPs) synthesised by the RPE, types 1, 2, and 9. These MMPs are important in BrM homeostasis and its physiological function [43]. The catalytic activity of MMPs is regulated by tissue inhibitors of MMPs (TIMPs). TIMP-1 and TIMP-2 are thought to move freely within the BrM, while TIMP-3 is thought to be bound to the RPE and CEC basement membrane [44].

## 3. Ageing Processes in BrM

BrM undergoes significant structural changes with ageing, particularly in terms of its permeability [28].

### 3.1. Anatomical Changes

**Figure 2:**
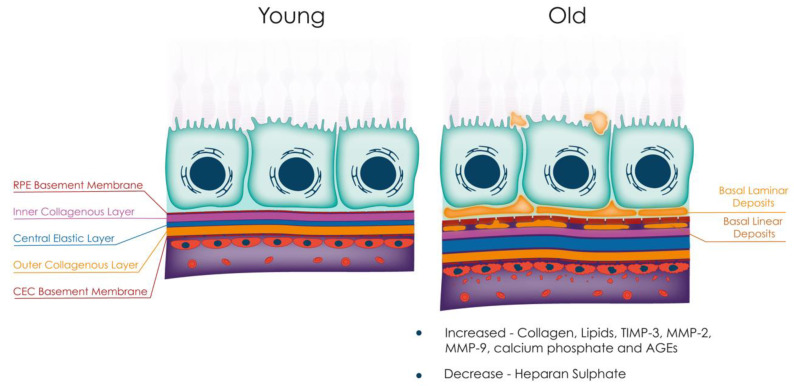
Age-related changes to BrM. With increasing age, BrM undergoes several alterations, including overall thickening due to increased deposition of collagen, lipids, TIMP-2, MMPs 2 and 9, calcium phosphate, and AGEs. Additionally, basal laminar and basal linear deposits are observed with age, and there is a reduction in heparan sulphate. These changes to BrM contribute to the pathogenesis of several retinal diseases, including age-related macular degeneration. Abbreviations: TIMP, tissue inhibitor of metalloproteinase; MMP, matrix metalloproteinase; AGEs, advanced glycation end-products; RPE, retinal pigment epithelium; CEC, choroidal endothelial cell.

BrM undergoes thickening with ageing [29,45,46,47], detectable using modern ophthalmic imaging (Figure 2). This phenomenon is likely due to the accumulation of lipids, including esterified cholesterol, fatty acids, and triglycerides [48,49], which reduces its permeability [50,51]. Changes in matrix molecules, such as an increase in MMPs 2 and 9 and their inhibitor TIMP-3, may also contribute to BrM thickening [43,52,53], as may advanced glycation end-products (AGEs) such as pentosides and carboxymethyllysine that cause inflammation via activation of AGE receptors on RPE and immune cells [54]. Changes in BrM laminin and proteoglycans, essential for its structural properties and RPE cell attachment, also occur with age [16]. RPE synthesises laminin 1 (α1ß1γ1), 5 (α3ß3γ2), and 10/11 (α5ß1/2γ1), allowing adhesion to BrM via integrin-mediated mechanisms [16]. However, heparan sulphate proteoglycans (HSPGs), key binders of the complement regulator Factor H (FH), have been shown to be reduced with age [55] and may lead to increased complement activation [56,57]. This may exacerbate the effect of the FH p.Y402H polymorphism (a major genetic risk factor for AMD) as it has been proposed that the variant protein binds less well to HSPGs than the wild-type protein [56,57].

### 3.2. Biomechanical Changes

Calcification with calcium phosphate deposition is also detected in the CEL with ageing [58], causing BrM to become stiffer. The elasticity of BrM has been shown to decrease linearly from as early as 21 years of age at a rate of approximately 1% per year [59], although this does not appear to be exaggerated in AMD [59].

### 3.3. Permeability Changes

The movement of water across BrM, as measured with Ussing chambers [60,61,62,63], decreases with age. The loss of water permeability is greatest in the ICL, particularly in the macular area as compared to the retinal periphery [62]. With age, there is also a reduction in the transport of protein molecules. One study observed an almost 100% reduction in the permeability of serum proteins across BrM from the first to the ninth decades of life (from 3.5 × 10^−6^ to 0.2 × 10^−6^ cm/s) [64]. The study further observed that in younger donors, BrM was permeable to serum proteins with molecular weights greater than 200 kDa, while in elderly donors, the threshold decreased to 100 kDa [64]. Nevertheless, these models may not fully capture the complexity of human physiology. For instance, recent research has indicated that some complement molecules with a molecular weight of less than 100 kDa cannot penetrate BrM, implying that there may be additional factors (such as sample preparation, pH, temperature, and osmolarity) that could influence the outcomes of such experiments.

Table 1 provides an overview of functional and anatomical changes in BrM associated with ageing, while Table 2 summarises the changes in the composition of each layer of BrM with age.

## 4. Overview of AMD

AMD classification has traditionally been based on anatomical changes observed through ophthalmic imaging, with early, intermediate, and advanced (or late) stages being distinguished. While early/intermediate forms have been associated with functional changes such as impaired dark adaptation, advanced AMD has been the primary focus of research due to most visual impairment being caused by this stage. Advanced AMD can be broadly classified into two clinical forms: neovascular AMD and geographic atrophy [45]. In neovascular (or exudative) AMD, subretinal neovascularisation develops below or above the RPE. These abnormal blood vessels often leak serous fluid or blood, resulting in macular oedema and/or haemorrhages that can lead to rapid visual loss. Repeated disruption of retinal architecture in this manner may also result in profound, irreversible vision loss. In geographic atrophy due to AMD, a zone of photoreceptor, RPE, and CC degeneration typically starts within the macula and expands to involve the fovea. It has recently been estimated that 16% of patients with bilateral geographic atrophy are registered severely sight impaired within an average of six years [4].

**Figure 3:**
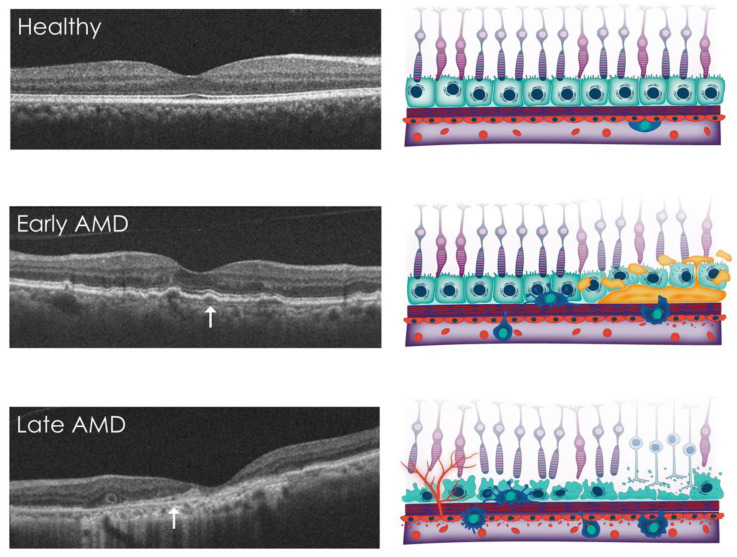
Pathological changes at the BrM interface in AMD. In healthy individuals (**top panels**), the retinal tissues are in a state of metabolic equilibrium. Early/intermediate AMD (**middle panels**) is characterised by the accumulation of extracellular deposits (illustrated in orange), such as soft drusen/BLinDs beneath the RPE and subretinal drusenoid deposits above it. These deposits mainly consist of lipoproteins of dietary and photoreceptor outer segment origin and are excreted by the RPE. They accumulate in the sub-RPE space and impede transit to the choroid due to ageing of the Bruch’s membrane. The accumulation of these deposits is associated with underlying choriocapillaris vascular dropout and initiates an immune response that leads to the recruitment of inflammatory cells. These deposits increase the risk of advanced stages of AMD, including atrophy and neovascularisation (**bottom panels**). Notably, BrM is more visible in areas of RPE separation and choroidal neovascularisation (white arrows). Changes within the Bruch’s membrane are not depicted in this figure. Open-source images used with permission [88]. Abbreviations: AMD, age-related macular degeneration; BLinDs, basal linear deposits; BrM, Bruch’s membrane; RPE, retinal pigment epithelium.

As the advanced subtypes of AMD are considered irreversible, it is important to understand the processes at early and intermediate stages of the disease to identify patients benefitting most from emerging therapies. Both advanced AMD subtypes are typically preceded by the accumulation of extracellular material in the BrM of the central retina. These are primarily termed drusen, focal deposits of proteins and neutral lipids such as esterified cholesterol, with apolipoproteins B and E located between the basal lamina of the RPE and the inner collagenous layer of BrM [65,66] (Figure 3). Of over 100 proteins identified in drusen, the commonest include vitronectin and clusterin (which both participate in the control of the lytic activity of complement), with TIMP-3, serum albumin, and crystallins [67] also identified—several of which have undergone oxidative protein modifications [68]. Two additional forms of BrM thickening are also observed in early disease, namely basal laminar deposits (BLamDs) and basal linear deposits (BLinDs), which are appreciable as diffuse thickening of the inner aspect of BrM on imaging. These are distinguished by their anatomical location: BLamDs are found above the RPE basement membrane, while BLinDs are located beneath this, adjacent to the BrM ICL. BLamDs also have characteristic staining patterns and may have a striated histological appearance due to long-spacing collagen [69,70]. These deposits have similar lipid and protein constituents to drusen [71] and are often confluent with them [70]. Collections of extracellular material in the subretinal space between the apical RPE and photoreceptors, known as subretinal drusenoid deposits or reticular pseudodrusen, have also been observed and are considered a high-risk phenotype for AMD progression [72].

### 4.1. Genetic Risk Factors

AMD is a complex disease resulting from the interplay of both genetic and environmental factors. Its strong genetic component, with an up to 10-fold increase in risk when a parent or sibling is affected, makes it one of the most heritable complex diseases [73,74]. Indeed, a large twin study has proposed heritability (additive genetic) estimates of 46% to 71% for AMD severity classification based on clinical examination and colour fundus photography [75]. The genetic basis of AMD has been widely studied through genome-wide association studies (GWASs). These studies identify various genetic variants associated with increased risks of developing AMD, including a common single nucleotide polymorphism (SNP) in the *CFH* gene (*CFH* p.Y402H) conferring an approximately two-fold higher risk of developing late-stage AMD per allele [76] and a common haplotype affecting the age-related maculopathy susceptibility 2 (*ARMS2*) and HtrA serine peptidase 1 (*HTRA1*) gene loci [77]. Subsequent GWASs have detected 52 independent and rare variants distributed across 34 loci that account for more than half of the condition’s heritability [78]. These include many genes involved in the alternative pathway of complement activation (*CFH, CFI, CFB*, *C2*, *C3, C9*), as well as genes involved in cholesterol metabolism (*ABCA1*, *APOE, LIPC*), matrix remodelling (*TIMP3*, *COL8A1*), and other functions [78,79,80]. Both common and rare variants have been found to contribute to AMD risk, with rare variants in *CFI* associated with particularly high risk [81,82]. Further research is necessary to fully understand the role of these genetic variants in the development and progression of AMD.

### 4.2. Environmental Risk Factors

In addition to ageing, smoking is a significant environmental risk factor for AMD, with the odds ratio for current smokers compared to ex-smokers being estimated by one meta-analysis to be 1.78 (95% confidence intervals, 1.52–2.09) [73]. Smoking can increase inflammation and oxidative stress in the RPE, which can be further exacerbated by changes in the choriocapillaris such as abnormal blood vessel growth and vasoconstriction [83,84]. Large longitudinal studies have suggested that smokers remain at high risk of developing neovascular AMD up to 20 years after smoking cessation [85,86], although these analyses were not adjusted for socioeconomic status. The literature on possible associations of AMD with diet, sunlight exposure, and cardiovascular or metabolic disease is inconsistent, partly due to difficulties in quantifying or standardising these factors. Antioxidant vitamin and mineral supplementation may delay late AMD progression in some high-risk individuals, although this evidence is of low certainty [87].

## 5. Non-Invasive Imaging of BrM

Advances in ophthalmic imaging, particularly optical coherence tomography (OCT) and OCT-angiography, have improved visualisation of BrM in high resolution, allowing age-related changes and retinal pathologies such as AMD to be studied.

**Figure 4:**
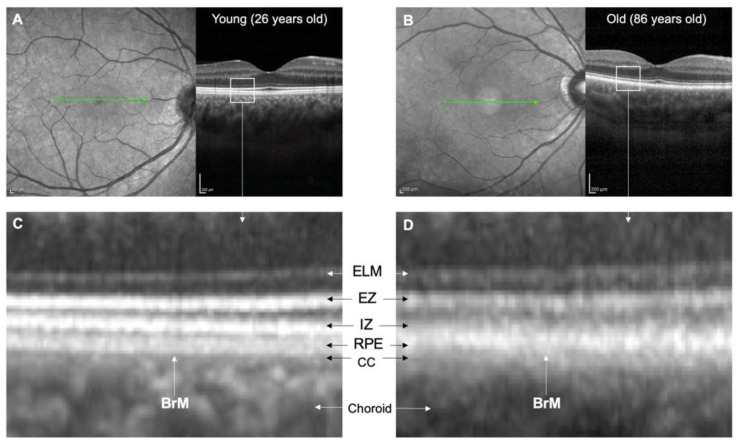
Comparison of OCT images of younger and older retinas. (**A**,**B**) show scanning laser ophthalmoscope fundal images and corresponding horizontal 15-degree OCT line scans of a 26-year-old female with a clear lens and an 86-year-old female who had undergone cataract surgery. The OCT images were averaged from 100 B-scans. (**C**,**D**) show high magnification views of the outer retina. In the 26-year-old individual, clear distinctions can be made between the IZ, RPE, BrM, and CC. However, in the 86-year-old individual, these structures are indistinct, and there is apparent thickening of BrM and thinning of the CC. Abbreviations: BrM, Bruch’s membrane; CC, choriocapillaris; ELM, external limiting membrane; EZ, ellipsoid zone; IZ, interdigitation zone; OCT, optical coherence tomography; RPE; retinal pigment epithelium.

BrM, in conjunction with the RPE, is known as the RPE–BrM complex, identified as the fourth outer hyper-reflective band of the retina on commercially available spectral-domain or swept-source OCT devices [89]. In young adults, the interdigitation zone, RPE, BrM, and choriocapillaris can be distinguished; however, with age, their demarcation becomes less clear due to BrM thickening and BLamDs (Figure 4). Separation of the RPE and BrM due to choroidal neovascularisation (CNV), drusen, or RPE atrophy can sometimes be seen as a thin hyper-reflective line on OCT [90] (Figure 3). The increased thickness of the RPE–BrM complex with age may also correlate positively with quantitative autofluorescence [91]. As previously described, the BrM thickening is partly attributed to age-related accumulation of lipoprotein-related lipids that form basal linear deposits (BLinDs) and soft drusen. These deposits are not visible on OCT but may appear as scattered hypofluorescent spots on late-phase indocyanine green angiography [92].

On OCT-angiography, BrM appears as a dark (hypoechoic), avascular band. In cases of type 1 and type 2 macular neovascularisation, OCT-angiography may show a signal crossing through the BrM [90]. However, it is important to note that projection artifacts can sometimes cause large retinal vessels to appear at the level of the BrM [90].

### 5.1. In Vivo Imaging of BrM in AMD

Eyes with early and/or intermediate AMD have a thicker RPE–BrM complex than healthy eyes according to global and/or low-density OCT spatial analysis [92,93]. The central macula shows the most significant thickening [94]. High-density OCT thickness analysis has shown that the central RPE–BrM complex is thicker in intermediate AMD than early AMD, likely due to the build-up of lipoprotein-related lipids [95]. The thickness of the RPE–BrM complex, outer plexiform, and outer nuclear layers decreases with increasing eccentricity from the foveal centre, which may relate to the loss of rod photoreceptors in AMD [95]. Recent data suggest that the thickening of the RPE–BrM complex in AMD eyes is mainly due to the accumulation of BLamDs and extracellular matrix material between the basal lamina and plasma membrane of the RPE, rather than accumulation within the RPE or BrM itself [69]. This can appear as a “double-layer sign” or splitting within the RPE–BrM complex on OCT [69], which is an emerging biomarker of neovascular AMD [96].

### 5.2. In Vivo Imaging of BrM in Inherited Retinal Degenerations (IRDs)

Thickening of BrM can also be seen in IRDs such as dominant drusen (DD), late-onset retinal degeneration (L-ORD), pseudoxanthoma elasticum, and Sorsby fundus dystrophy (SFD) [97]. OCT scans show a separation of the RPE and BrM, appearing as two distinct hyper-reflective bands in DD, L-ORD, and SFD, which has been suggested as a potential biomarker for these degenerative conditions [97]. The extent of separation of the RPE–BrM complex has been suggested to correspond with the degree of visual symptoms in DD and SFD [97]. This separation is thought to be caused by the accumulation of BLinDs and BLamDs between the elastic lamina of BrM and the visible apical RPE, where melanosomes are concentrated [97].

## 6. Overview of Complement

**Figure 5:**
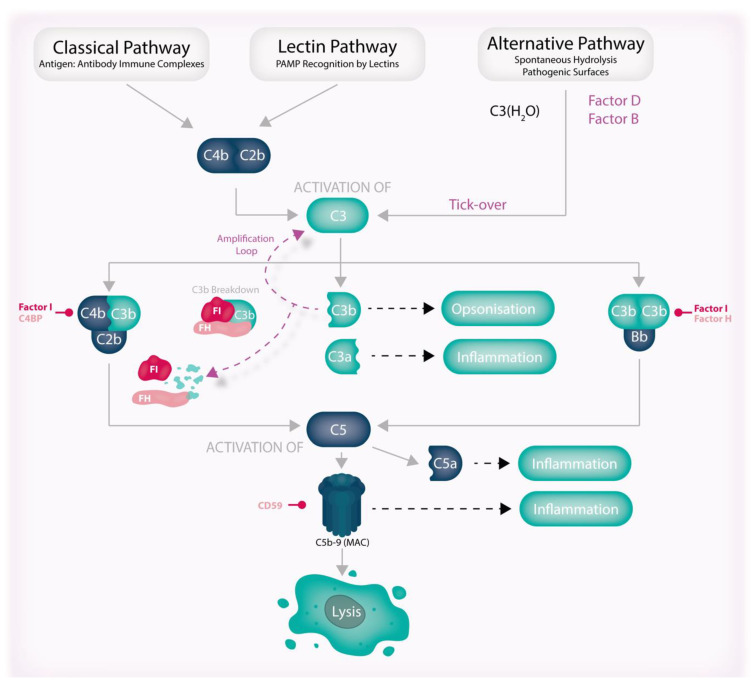
The pathways of the complement system. The complement system is a network of proteolytic pathways that work together to maintain the body’s homeostasis. The alternative pathway is continuously activated through the hydrolysis of C3 to C3(H_2_O) in a process known as “tick-over” and triggers the formation of C3 convertases (C4b:C2b, C3b:Bb (not shown)) and C5 convertases (C4b:C2b:C3b and C3b:Bb:C3b), leading to the production of anaphylatoxins, effector molecules, and the C5b-9 complex (MAC). Although it causes significant inflammation, cell lysis as a result of the MAC is a rare occurrence. Importantly, the production of C3b results in further C3 activation through the formation of the C3b:Bb convertase (also not shown) in a process known as the amplification loop. These pathways are regulated by various factors and cofactors that interact with complement components, activating enzymes, or each other. FH plays a critical role as a cofactor for FI by binding C3b and possessing cofactor/decay-accelerating activities that inhibit the alternative pathway amplification loop and initiate the breakdown pathway of C3b. Abbreviations: FH, Factor H; FI, Factor I; MAC, membrane attack complex; PAMP, pathogen-associated molecular pattern.

The complement system is a complex network of approximately 30 proteins that plays a vital role in the immune response. There are three main pathways: the classical pathway, the lectin pathway, and the alternative pathway (Figure 5). The complement system comprises both activating and controlling proteins that are found throughout the body in individual or complex forms. Complement represents a significant 15% of the globulin fraction of blood and is responsible for lytic activity against invading pathogens as part of the innate immune system [98]. However, recent evidence also indicates that complement has critical roles in adaptive immunity, cell senescence, and tissue remodelling [7]. Complement activation occurs through a process called “tick-over”, in which the central component, C3, activates at a low level in plasma and initiates the alternative pathway (AP). This sets off a cascade of protein interactions that culminates in rapid inflammation and cell lysis via the formation of a pore on membranes of a bacterium or antibody-coated cell surface, known as the membrane attack complex (MAC) [7,99]. The primary impact of the MAC on nucleated cells is the induction of inflammation through multiple signalling pathways and activation of the NLRP3 inflammasome. Despite its potent pro-inflammatory properties, The MAC rarely leads to the lysis of nucleated cells due to the presence of ion pumps and endo/exocytotic mechanisms that prevent cellular damage [100]. These findings underscore the importance of the complement system in maintaining immune function and preventing disease.

The complement system’s inability to distinguish self from non-self can lead to unintended consequences on healthy tissues in the vicinity of activation, resulting in local cell dysfunction, damage, or even death. While regulatory proteins normally coat our own cells and quickly inactivate activating proteins and complexes to control complement activation, even brief episodes of activation can contribute to disease progression over time.

## 7. Complement Dysfunction in AMD

### 7.1. The Role of Complement in Choroidal Homeostasis

The relative importance of the RPE and CC in the development of AMD is a subject of ongoing debate, but it is clear that both tissues play a role [101,102]. Early in the disease process, there is a vascular loss of CC that corresponds to the presence of drusen [103]. In AMD and normal ageing, the accumulation of MAC on CC endothelium also increases in a process that is exacerbated by the presence of the *CFH* p.Y402H genotype [104,105,106]. This can result in the death of choroidal endothelial cells in culture [105,106,107,108,109,110,111]. Complement may play a constitutive role in the choroid and retina decades before the onset of AMD, as indicated in human tissue studies by higher MAC deposition [108,112], CD59 expression [113], and FH production relative to other retinal tissues. Thus, complement may have a significant impact on choroidal homeostasis through its effects on modulating macular neovascularisation and immune cell regulation.

### 7.2. The Role of Complement in RPE Function

Under normal conditions, RPE cells express complement proteins that facilitate the opsonisation and phagocytosis of photoreceptor outer segments and waste products of the visual cycle through a process known as autophagy [114,115]. This is supported by the presence of multiple complement components and regulators in drusen [67,114,116,117,118]. In fact, complement proteins C3 and Factor B (FB) have been found to be essential for drusen formation in mouse models of inherited retinal degeneration [119,120,121]. This model is triggered by inducing the missense p.R345W variant in the *EFEMP1* (EGF-containing fibulin-like extracellular matrix protein 1) gene, which encodes fibulin-3 involved in extracellular matrix development. This variant causes Doyne honeycomb retinal dystrophy in humans, which is characterised by early-onset macular and peripapillary drusen. In mice, this variant recreates many of the histological features of AMD, such as the accumulation of coalescing, electron-dense sub-RPE debris and general RPE and choroidal abnormalities, including degeneration, vacuolation, loss or disruption of RPE basal infoldings, choroidal atrophy, and focal thickening and invasion of cellular processes into BrM. Despite this, it is important to acknowledge the limitations of using murine models to study AMD, as has been explored elsewhere [122].

Under normal conditions, complement expression in the RPE and neural retina is generally low compared to the choroid [114], but this may increase under conditions of stress or stimulation. In ageing and AMD, there is increased expression of the complement protein C3 within RPE cells and on their basal membranes [114,123]. This is also seen in mice, where increased AP activation in the RPE/choroid has been identified [124]. Exosomes secreted by stressed RPE cells have also been found to be coated with complement components [125,126]. The role of this increased complement expression in the RPE is not yet clear, as it could either be protective or contribute to disease pathogenesis. To protect themselves from harmful complement activation, RPE cells secrete FH and express several regulators of the complement system, such as CD59 on their apical side [113,127,128] and CD46 (also known as membrane cofactor protein or MCP) on their basal side [113,129,130]. CD59 inhibits formation of the MAC, whereas FH and CD46 drive the breakdown of surface-bound C3b. In addition to its role in regulating the complement system, FH also binds to pro-inflammatory lipid peroxidation products on the surface of damaged RPE cells and controls inflammation in their vicinity [131].

### 7.3. Impact of Complement Overactivation on RPE Function and Viability

Reduced expression of FH and CD46 by RPE cells is observed in AMD, and this coincides with increased deposition of the MAC and cell lysis [132,133]. Dysfunctional FH, including the p.Y402H variant, can also lead to overactivation of the complement system, which can impair the essential functions of RPE cells, including autophagy, lysosomal function, and energy metabolism. This has been demonstrated in studies using RPE cells derived from patients with AMD with high-risk complement genotypes [134,135,136,137]. RPE cells with impaired lysosomal function are thought to be susceptible to MAC deposition due to their inability to adequately recycle and traffic complement inhibitors such as CD59 to the cell surface [138]. A lack of functional FH may also impair the clearance of lipid peroxidation products [131], leading to oxidative stress [139]. Oxidative stress, in turn, may downregulate the expression of complement regulators by RPE cells [113,127,140,141,142] through apoptotic shedding and exosomal release of CD46 and CD59 [143]. Furthermore, infiltrating subretinal macrophages in AMD may induce complement dysfunction in RPE cells [144]. These interactions can create a vicious cycle of complement activation, oxidative stress, and cellular dysfunction.

Complement-mediated dysfunction of the RPE may contribute to the development of AMD. As previously discussed, this has been implied in a variety of mouse models of inherited retinal degeneration, in which the presence of drusen-like deposits is dependent on complement for formation [119,120,121,145]. Complement activation leading to MAC deposition also has immunomodulatory effects on nucleated cells and stimulates the RPE to produce metalloproteinases and VEGF [146,147], which may drive macular neovascularisation, thus suggesting that complement overactivation may lead to both exudative and non-exudative AMD. In addition, our recent study of the UK Biobank, the largest repository of retinal imaging data to date, found that the common at-risk FH genotype (*CFH* p.Y402H) and different rare FI variants were associated with reduced RPE–BrM complex thickness in healthy participants, suggesting that complement overactivation may have a negative impact on the function and viability of RPE cells [81]. These effects may be more pronounced with advancing age or disease.

To protect against complement activation and cellular dysfunction, overexpression of secreted and membrane-bound complement regulatory proteins has been suggested as a therapeutic strategy [111,148,149,150,151].

### 7.4. Complement Dysfunction in Macular Neovascularisation

Several experiments using mouse models of laser-induced CNV have demonstrated that all complement pathways, particularly the alternative and terminal pathways, play a significant role in angiogenesis [150,152,153,154,155,156,157,158,159,160,161,162,163]. The alternative pathway is responsible for the majority of C5a and MAC formation [164], which suggests that the terminal pathway may be causative in CNV formation. Additionally, several studies have highlighted the potential role of the vascular endothelial growth factor (VEGF) pathway in mediating these effects. VEGF is an angiogenic protein that exerts its effects primarily through VEGF receptor 2 (VEGFR2). For example, sublytic levels of MAC have been shown to stimulate the release of growth factors from nucleated cells [99,100], including VEGF from RPE in cell culture studies [146,165]. In addition, the inhibition of CD46 by gene knockout in mouse models led to increased VEGF production in the retina and choroid, increased MAC deposition on these tissues, increased susceptibility to laser-induced CNV, and degenerative changes in retinal tissues resembling an AMD phenotype [166,167]. It is worth noting that the VEGF–complement interaction may also be reciprocal; VEGF inhibition has been shown to increase complement activation by decreasing FH expression in RPE and renal podocytes through VEGFR2- and PKC-α/CREB-dependent signalling [168].

The activation of complement can have varying effects depending on the stimulus or location in the body. For instance, the anaphylatoxins C3a and C5a have been found to enhance laser-induced CNV in some cases, but may have anti-angiogenic properties in mouse models of retinal hypoxia through the stimulation of mononuclear phagocytes to secrete soluble VEGF receptor 1 that inhibits VEGF [169,170]. There is also conflicting evidence on the role of FB, a serine protease involved in the alternative pathway of complement activation, in angiogenesis. Some studies have shown that FB has a pro-angiogenic effect mediated through increased VEGF and VEGFR2 expression by endothelial cells in mouse models of laser-induced CNV and retinal hypoxia [81,153,171]. MMP expression in choroidal endothelial cells has also been found to increase with MAC-dependent complement activation [172], suggesting that complement may promote angiogenesis through multiple pathways. In contrast, other research has shown that inhibiting FB in a mouse model of retinal hypoxia potentially leads to increased neovascularisation, as well as increased severity and duration of retinopathy, through a reduction in endothelial cell apoptosis and increased expression of CD55 [163], a regulator of complement. These findings suggest that complement may have complex and diverse roles in angiogenesis, and further research is needed to fully understand its mechanisms of action.

Understanding the complex interplay between complement and angiogenesis has significant implications for the development of effective and safe complement-based therapies. The balance of complement activation is crucial, as both underactivation and overactivation can have adverse effects. However, the effects of complete inhibition of complement in the eye remain unclear and require further exploration. For example, it is unclear why the development of macular neovascularisation is a possible emergent adverse event of intravitreally administered complement inhibitors for dry AMD and whether this is due to treatment modality (e.g., the PEGylation of agents such as pegcetacoplan and avacincaptad pegol) or target [173]. The complexity of this field is further compounded by limited evidence from case reports suggesting that intravitreal inhibition of VEGF may lead to disorders of complement overactivation in the kidneys [174]. Newer agents that target both complement and the VEGF pathways, such as Ranifitin (manufactured by *Apellis*, Waltham, MA, USA), may address these safety concerns. However, the role of VEGF secreted by the RPE in maintaining the choriocapillaris is still not fully understood [175]. Some studies have found a correlation between intravitreal anti-VEGF therapy for neovascular AMD and an increased risk of developing GA [176,177,178,179], but it is unclear if this is due to common advanced AMD processes or if VEGF inhibition leads to retinal cell damage related to complement. There is also debate over whether non-exudative macular neovascularisation can support photoreceptors and the RPE in GA [180,181,182]. Further research into the biological interactions between complement and VEGF will be important for guiding drug development and clinical trial design [183].

### 7.5. Immune Cell Regulation

Complement plays a vital role in directing the cellular immune response to diseases, largely explored in cancer and sepsis but also relevant for AMD [184].

A critical component in this area is the anaphylatoxin C5a, which plays multiple roles in immune regulation. While it has well-known pro-inflammatory functions mediated through its C5aR receptors and activation of the NLRP3 inflammasome (as previously reviewed [185,186]), it is also essential for the development and regulation of T-cell immunity [187,188,189,190,191,192,193]. C5a signalling generally leads to inflammation through the protection of CD4+ T-cells against apoptosis and the upregulation of pro-inflammatory cytokines such as IL-17 and IL-22 [187]. Furthermore, in the choriocapillaris, C5a upregulates the expression of leukocyte adhesion molecules on endothelial cells [109] and plays a key role in recruiting γδ T-cells [194], a specialised class of tissue-resident lymphocytes that rapidly expand in response to stress induced by pathogens or endogenous stimuli and initiate neutrophil recruitment, phagocyte activation, and granuloma formation [195].

It has also been shown that MAC recruits and activates leukocytes by coordinating inflammasome activation and endothelial cytokine signalling [196,197,198,199,200], which may initially preserve tissue function by rapidly suppressing danger; however, sustained production of reactive oxygen species, proteases, and inflammatory cytokines can contribute to dysfunction of retinal structures [6,201]. With increasing age, endothelial cells may become more sensitive to MAC injury due to higher cytoskeletal Rho kinase activity [202]. In a series of experiments, Calippe and colleagues showed somewhat surprisingly that FH prevents the clearance of subretinal mononuclear phagocytes through CD47-dependent mechanisms [203]. Interestingly, the common FH risk variant in AMD, *CFH* p.Y402H, resulted in even poorer microglial elimination [203]. These findings suggest that non-resolving subretinal inflammation due to complement may be exacerbated by genetic predispositions.

## 8. Local vs. Systemic Complement Production in AMD

The debate over the role of systemic versus local complement activation in AMD is highly relevant to drug design and delivery [204]. While most complement proteins are produced in the liver, others (such as C1q, C7, Factor D, and properdin) are produced at extrahepatic sites by various cells, including monocytes, macrophages, and glial and endothelial cells [7,204,205]. Local complement synthesis is regulated by cytokines and growth factors [206], and local biosynthesis is responsible for downstream complement effects in some tissues. Emerging evidence suggests that the effects of complement activation vary across tissues based on their structural properties, with some tissues being more susceptible to local activation and others being more prone to systemic activation. For example, in experimental mouse models, the renal tubular epithelium has been found to be more susceptible to donor kidney-derived C3, while the fenestrated capillaries of the renal glomerulus are more prone to systemic C3 activation [204,207]. This concept may be particularly relevant in the context of the kidney and eye, both of which have structural and developmental similarities [208] and are known to be susceptible to complement dysregulation [7,209]. These findings suggest that targeted complement inhibitors may be more effective in certain tissues at improving disease outcomes.

The BrM is believed to play a crucial role in partitioning complement activation into local or systemic pathways within the eye. As previously mentioned, its physical, electrostatic, and biochemical properties contribute to this function [14]. As noted previously in Section 2, BrM permeability declines with age [64]. Using donor eye tissue, Clark and colleagues found that certain complement proteins, such as C3/C3b, FH, FB, and FI, are unable to penetrate the BrM even at small quantities, while others, such as Factor D, Factor H-like protein 1 (FHL-1), and C5a, can cross the BrM but at reduced concentrations [35]. The fact that FHL-1 (49 kDa), a splice variant of the *CFH* gene, has better tissue penetration than the full-length, glycosylated FH protein (155 kDa) suggests that it may play a specialised role in controlling complement activation locally [35,36]. However, FHL-1 is not as effective at inhibiting AP overactivation as FH, as it has reduced binding to extracellular matrix proteins and cannot alter its conformation to conceal its catalytic domains when not bound to host structures [210]. Another clue that FHL-1 may have evolved specifically to control complement activation locally is that the genetic sequences consistent with FHL-1 expression are found in old world monkeys, but not in their new world ancestors [210]. Beyond FHL-1, it has also been shown that FI is found at significantly lower levels in the aqueous fluid of the eye compared to FI levels found in simultaneously sampled serum [211]. This finding provides further evidence for the concept that complement regulation in the eye is compartmentalised and distinct from systemic regulation. These results have important implications for the delivery of complement therapeutics across the BrM and should be considered in drug design and development.

There is evidence suggesting that levels of circulating complement activation products may be associated with the severity of AMD, although it is not yet clear whether this is a causal relationship or simply a result of ageing [212,213,214,215,216,217]. Systemic inflammation and endothelial dysfunction have been linked to reduced chorioretinal thickness [218], which may precede the development of AMD. Additionally, drusen, a hallmark of AMD containing systemic proteins, have been suggested to result from pathological transport of visual cycle by-products across the capillary wall or BrM [219]. However, patients with genetic abnormalities in the complement system tend to only develop overt disease in one organ (such as AMD, C3 glomerulopathy, or atypical haemolytic uraemic syndrome), even in the presence of rare, pathogenic variants or haploinsufficiency of FH and FI [81,220,221,222,223]. In a multicentre study of liver transplant patients, incident AMD was found to be associated with recipient, but not donor, *CFH* p.Y402H status, suggesting that systemic complement production by the liver may not contribute to retinal disease [224]. These findings suggest that the end-organ response to complement overactivation may be influenced by genetic determinants at a tissue level, which have yet to be fully mapped.

## 9. Complement as a Therapeutic Target

**Figure 6:**
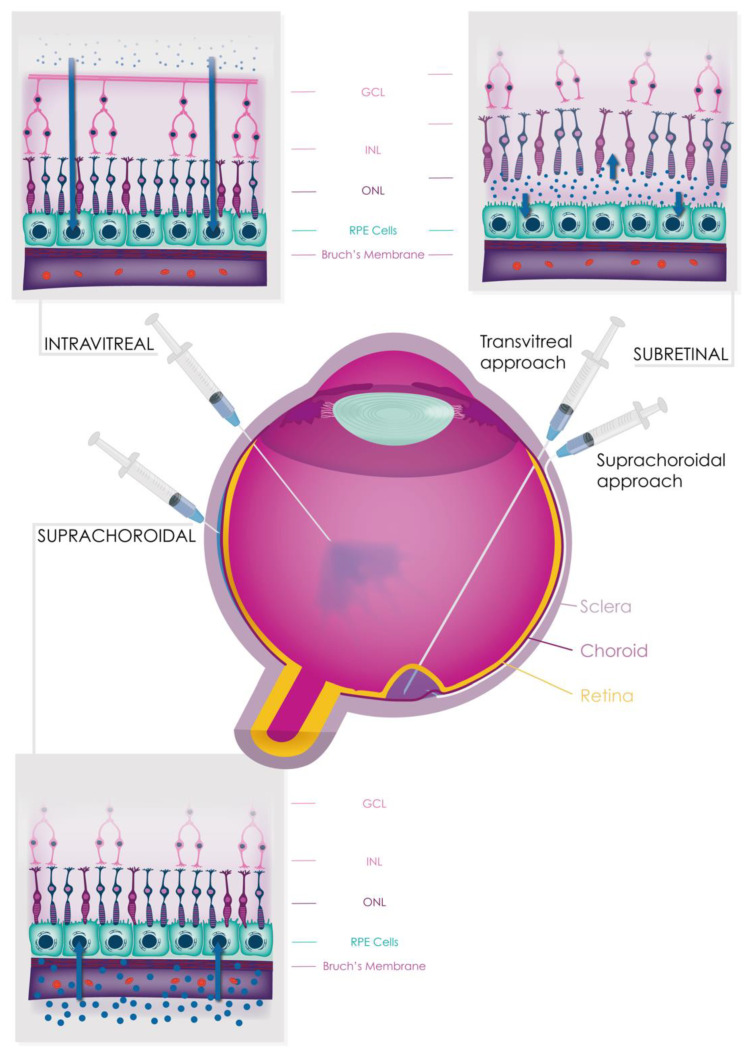
**Figure 6**. Different types of drug delivery route in the human eye. Intravitreal (IVT) injections are a common method of administering therapeutic agents to the eye. Other routes of administration, such as subretinal and suprachoroidal injections, are also being investigated. Subretinal agents may be administered transvitreally or by cannulation through the suprachoroidal space. Viral gene therapies targeting the retina vary in their vector distribution, retinal tropism, and transduction efficiency depending on the route of delivery used. While efficacy is a primary focus, safety is also a key consideration, as it relates to inflammation and iatrogenic complications. Abbreviations: GCL, ganglion cell layer; INL, inner nuclear layer; ONL, outer nuclear layer; RPE, retinal pigment epithelium.

Despite facing numerous challenges in the past, such as a high plasma concentration, high daily turnover, and a volatile nature, the field of complement inhibition has made significant progress in recent years [225]. This is due in part to a better understanding of the genetic and functional roles of complement in various tissues, as well as improved structural information regarding the proteins involved. As a result, a number of complement inhibitors are currently in late-stage clinical trials for the treatment of AMD, with the first such agent (pegcetacoplan, manufactured by *Apellis*, Waltham, MA, USA) recently receiving approval from the U.S. Food and Drug Administration (FDA) for the treatment of GA [173]. Complement therapies can be delivered systemically or directly to the eye by intravitreal, subretinal, and suprachoroidal injections (Figure 6). These therapies are diverse in their design and delivery, though it remains to be seen which strategy will be most effective for ophthalmic indications. Given the potential for complement activation to contribute to the development of AMD and other conditions, inhibiting the alternative pathway of complement has garnered significant interest as a therapeutic strategy [226].

### 9.1. Systemic Therapies

Systemic complement therapy, which involves delivering drugs orally, subcutaneously, or intravenously, is appealing due to its ease of administration. Additionally, the high blood flow in the choroid makes it possible for these therapies to quickly reach their target tissue [227]. However, as mentioned previously, although choroidal vessels are highly permeable, BrM is less so, making the diffusion of systemic therapies through it a challenge. The blood–retinal barrier (BRB)—comprising the tight junctional complexes between retinal capillary endothelial cells (inner BRB) and retinal pigment epithelial cells (outer BRB)—may also impede the diffusion of molecules from the systemic circulation to the retina. The inner BRB is only permeable to molecules with a small diameter or molecular weight (i.e., up to 2 nanometres or a few hundred daltons) [228], while the outer BRB is also highly selective, allowing hydrophilic and larger molecules to cross at a slower rate than their small, lipophilic counterparts [229]. However, as mentioned earlier, the relevance of diffusion chamber experiments to human health remains uncertain. Despite these challenges and limitations, it is possible that if complement-mediated choroidal dysfunction is a major contributor to AMD, saturating this tissue with systemic therapies may be sufficient to improve the disease.

While systemic administration of drugs has the potential to provide benefits in the treatment of posterior segment conditions, there is a lack of information about drug distribution in this area. Studies have simulated drug distribution from the plasma to the vitreous in rabbits [230], but it is unclear how these pharmacokinetics translate to humans and whether higher rates of systemic drug clearance may be a factor [228]. Other considerations for systemic administration include the effects of protein binding in the plasma on drug permeability, the potential for transcellular diffusion to bypass the tight junctions of the BRB and increase drug permeability [228], and the risk of systemic toxicity. Complement inhibition, in particular, has been associated with an increased risk of encapsulated bacterial infection and the loading of host cells with complement proteins upstream of the inhibited molecule [231,232], resulting in a need for vaccination and the exclusion of patients who are immunocompromised. Nevertheless, small molecule therapeutics such as the FDA-approved avacopan (an inhibitor of C5a Receptor 1, previously developed by *ChemoCentryx*, San Carlos, CA, USA, now by *Amgen*, Thousand Oaks, CA, USA) and iptacopan (also known as LNP023, an inhibitor of FB, developed by *Novartis*, Basel, Switzerland), which has recently achieved its Phase 3 outcomes, have shown that systemic complement inhibition can safely treat systemic conditions such as paroxysmal nocturnal haemoglobinuria [233,234]. Homing agents that bind to neoepitopes on complement proteins or stress-related antigens on apoptotic or necrotic cells may offer an additional way of restricting drug action to inflammation hotspots and reducing the risk of widespread systemic effects [225], though it is unclear how such a therapy would specifically target the eye.

To date, intravenous eculizumab (manufactured by *Alexion Pharmaceuticals*, New Haven, CT, USA), a monoclonal antibody against C5, is the only systemic complement inhibitor to have completed a randomised controlled trial in AMD, and it failed to show any significant benefits in terms of anatomy or function in a randomised controlled trial for GA [235]. Despite the limited duration of treatment (6 months) and small sample size with several methodological biases [173], its inability to demonstrate any clinical efficacy in terms of reducing GA progression or vision loss has raised questions about the usefulness of systemic complement inhibitors for AMD treatment. The results of Phase 2 clinical trials of several systemic agents are eagerly awaited and may provide more insight into the potential of systemic complement inhibitors for AMD [236]. These include IONIS-FB-LRx (developed by *Ionis Pharmaceuticals Inc.*, Carlsbad, CA, USA), a subcutaneous antisense oligonucleotide inhibitor of FB (NCT03815825, https://clinicaltrials.gov/ct2/show/NCT03815825), danicopan (formerly developed by *Achillion Pharmaceuticals*, CT, US, now by *AstraZeneca*, Cambridge, UK), an oral small molecule inhibitor of Factor D (NCT05019521, https://clinicaltrials.gov/ct2/show/NCT05019521), and iptacopan, the oral small molecule inhibitor of FB discussed above (NCT05230537, https://clinicaltrials.gov/ct2/show/NCT05230537). There are also many other systemic complement inhibitors in clinical development for other conditions [225].

### 9.2. Intravitreal Therapies

Intravitreal delivery of agents directly to the vitreous cavity of the eye is a commonly used approach for treating diseases of the central retina. This method has been particularly successful in the treatment of vascular retinal disorders such as neovascular AMD through the use of anti-VEGF agents. Intravitreal delivery is a relatively simple procedure that can be performed in an outpatient setting with minimal safety concerns [237]. Although achieving therapeutic levels with intravitreal formulations is challenging due to dilution in the vitreous and variable retinal penetration dictated by molecular characteristics, all of the complement inhibitors currently in late-stage clinical trials for AMD are being administered intravitreally, including pegcetacoplan, an anti-C3 agent, and avacincaptad pegol, an anti-C5 agent. In Phase 3 studies, these agents have shown anatomical benefits for the treatment of GA at one year, but it is still unknown whether they will meaningfully improve visual outcomes [173]. Furthermore, there are safety concerns regarding the induction of exudative AMD for both agents [173]. An interesting hypothesis relating to reduced C3 breakdown products on endothelial cell surfaces with a shift to a reparative proangiogenic macrophage phenotype has been proposed [238]. It is unclear whether this adverse event is related to class effects of complement inhibition or PEGylation, as discussed in Section 7.4, or other clinical factors such as the presence and activity of macular neovascularisation in the fellow eye of study participants. As such, longer-term studies following market authorisation will likely be needed to assess the real-world risks to patients. Pegcetacoplan has recently been granted FDA market authorisation for the treatment of GA due to its positive results in reducing GA progression for dosing up to 24 months. The submission of avacincaptad pegol to the FDA is anticipated to result in an outcome in Q3 2023. Both agents are still being evaluated by the European Medicines Agency (EMA).

Nevertheless, the frequent administration of these therapies (i.e., monthly or every other month) and the associated burden on healthcare systems and patients have led to a continued search for longer-acting treatments. One potential approach is the use of gene therapy to modulate complement expression in the retina over the long term. Gene therapy involves the delivery of nucleic acid cargo (such as DNA, mRNA, or small interfering RNA) using viral or non-viral vectors. In the retina, adeno-associated virus (AAV) vectors have shown promise as a gene therapy technology due to their effectiveness and ability to sustain gene expression for several years in certain IRDs [239,240,241]. While AMD is a complex genetic disease, advances in dual and triple AAV vectors with enhanced cargo capacity now allow for the delivery of larger genes [242], including anti-VEGF and complement regulatory molecules, which could potentially be used in gene therapy for AMD.

While intravitreal gene therapies have shown some efficacy in transducing inner retinal ganglion cells for the treatment of Leber hereditary optic neuropathy [243], transduction of photoreceptors in the outer retina has proven more challenging. The inner limiting membrane (ILM) and glial processes of the neural retina act as barriers to the diffusion of naturally occurring AAV serotypes in humans [244,245]. AAVs have a high affinity for extracellular matrix proteins such as heparan sulphate, which is found in both the ILM and BrM [244]. This affinity allows AAVs to home to the vitreoretinal interface and facilitates retinal transduction, though it also reduces their diffusion through the retina [246]. Transduction may be most effective at areas of thinner ILM, such as in the perivascular regions [247], resulting in variable effects across the retina. There have been attempts to manipulate the ILM to reduce the necessary viral load for effective transduction, including the use of proteases to digest the ILM [244,248,249], electric micro-currents [250], and non-penetrating AAV vectors to saturate heparan binding sites in the vitreous [251]. However, these techniques have not resulted in gene expression in the outer retina. Other experimental techniques, such as ILM peeling or vitrectomy [252,253], sub-ILM injection [254,255], and ILM photo-disruption with indocyanine green [256], have also been proposed, but their safety is uncertain.

One possible way to enhance outer retinal transduction through the ILM without further surgical intervention is to make amino acid substitutions in the viral capsid to reduce heparan affinity [246]. However, this approach may require higher vector concentrations to compensate for the long diffusion distance within the vitreous [247]. These higher doses may trigger a stronger immune response and increase titres of neutralising antibodies [257,258,259], which can spread through the blood and lymphatic tissues [260] and potentially lead to reduced transduction efficacy and a lack of transgene expression with repeated administration [261,262]. Additionally, triggering a systemic immune response against an AAV-based vector can result in unintended side effects such as transgene expression in lymphoid organs [263,264] or systemic complement activation [265,266]. The high prevalence and cross-reactivity of neutralising AAV antibodies [239] indicates that methods to mitigate anti-AAV immunity will be necessary before the safety and efficacy of high-dose intravitreal viral vector-based gene therapy can be fully evaluated [267]. Unfortunately, animal models may not always provide reliable data on this issue [268].

There is growing interest in the development of non-viral vectors, such as polymeric, liposomal, lipid, and inorganic vectors, due to concerns about the immunogenicity of viral vectors [269]. However, the anionic and composite properties of vitreal proteoglycans can also hinder the effectiveness of these agents [270]. Strategies such as PEGylation [271], charge modification [272], and coating with hyaluronate or chloroquine [273,274] have been shown to improve retinal transduction in animal models when administered intravitreally. However, more research is needed to fully understand the biological effects and potential adverse effects of delayed clearance of these approaches.

While there are numerous challenges to intravitreal gene therapy, transduction or transfection of the inner retina may be sufficient to treat AMD. Müller glia, which are the most abundant glial cells in the retina and play a multifaceted role in retinal homeostasis and response to injury, including potentially photoreceptor regeneration, may be a target for gene therapy [275,276]. Müller cells are also considered a major source of complement proteins in the inner retina [115,130,277]. However, it is unclear if Müller cell gliosis in advanced AMD could obstruct vector diffusion [278]. Early therapeutic intervention in the disease course may offer the greatest benefit [279], and there are several ongoing intravitreal gene therapy trials for advanced AMD [280], including JNJ-1887 (formerly AAVCAGsCD59), an intravitreal AAV-based vector expressing a soluble form of the MAC inhibitory protein CD59 that is being evaluated for the treatment of GA. In mice, JNJ-1887 has been shown to reduce laser-induced CNV formation beyond the site of vector delivery [150], indicating the potential superior coverage of intravitreal versus subretinal gene therapies. This investigational medicinal product has received Advanced Therapy Medicinal Product designation by the EMA and FDA fast track designation for dry AMD following positive Phase 1 safety signals announced in a recent press release (NCT03144999) (available online: https://www.jnj.com/janssen-announces-late-breaking-data-from-two-gene-therapy-programs-at-the-american-academy-of-ophthalmology-2022-annual-meeting (accessed on 2 April 2023)).

### 9.3. Subretinal Therapies

Subretinal administration is a method of delivering drugs to the retina that has been commonly used in IRD gene therapy trials [281]. In this approach, the subretinal space is approached transvitreally with a vitrectomy. The retina is then perforated via a fine retinotomy and surgically detached from the RPE through a subretinal injection, forming a transient subretinal “bleb” that lasts for less than 1–2 days [281]. This space has the advantage of being close to the target cell populations in the outer retina and having greater immune privilege compared to the vitreous [282]. Most AAV-based vector serotypes can be used to deliver genes to RPE and outer nuclear layer cells through the subretinal route [283], although transduction of other cell types may be variable [284]. Subretinal gene delivery has been shown to be more effective and have a faster onset than intravitreal delivery in animal models [260,285,286]. For example, subretinal, but not intravitreal, administration of a CR2–FH fusion protein has been shown to reverse smoking-induced RPE damage in a mouse model [286]. Subretinal delivery is also less likely to result in systemic exposure and, potentially, immunogenicity compared to intravitreal administration [260], and the effectiveness of subretinal gene therapy does not seem to be affected by the presence of neutralising antibodies in the host serum [287]. Ongoing phase 2 studies (NCT04437368 (https://clinicaltrials.gov/ct2/show/NCT04437368), NCT03846193 (https://clinicaltrials.gov/ct2/show/NCT03846193), NCT04566445 (https://clinicaltrials.gov/ct2/show/NCT04566445)) are exploring the potential of GT005 (developed by *Gyroscope Therapeutics Limited*, London, UK, a *Novartis* company), an investigational subretinal AAV-based vector expressing *CFI*, for the treatment of GA using two delivery methods (Figure 6).

Subretinal injections for viral gene therapy may trigger host immune responses [258,288,289,290,291], and the requirement for vitrectomy and transvitreal approaches can cause complications such as cataracts and retinal tear formation/detachment [292,293,294]. These issues may be partly addressed through the use of novel surgical delivery systems [295]. The subretinal space can be approached through the suprachoroidal space using a flexible conduit with transpupillary visualisation as it transits to the intended point of delivery. At this point, a microneedle is advanced into the subretinal space and the treatment delivered, avoiding the need for vitrectomy and its related complications. This method is being evaluated in the aforementioned GT005 trial using the Orbit^TM^ Subretinal Delivery System (NCT03846193, https://clinicaltrials.gov/ct2/show/NCT03846193). One potential limitation is that it forms a BrM perforation point, which in AMD eyes in particular could theoretically increase the risk of macular neovascularisation if positioned posteriorly near the fovea, although this has not been observed to date. Utilisation of more anterior delivery avoids this and would be appropriate for secreted protein gene therapy as with FI.

Increased activation of glial responses and chronic choroidal inflammation following subretinal, but not intravitreal, AAV vector administration has additionally been reported in primates [257]. Dose-dependent immune responses have also been observed, with high doses potentially leading to persistent inflammation and clinically significant vision loss in patients receiving high-dose AAV2 vector-based subretinal *RPE65* therapy [296]. Systemic steroids are often used to mitigate this phenomenon, but the long-term effects of this approach on the retina are unclear [281]. Moreover, subfoveal viral gene therapy has been associated with various adverse effects, such as RPE alterations, retinal thinning, foveal morphological changes, vision loss, and the variable duration of clinical effects [281,294,296,297,298]. ROCK inhibitors may improve synaptic remodelling and ameliorate these negative effects in the future [299]. Finally, the limited lateral diffusion of subretinal agents may not fully suppress complement activation in distal retinal sites [282]. Overall, while subretinal viral gene therapy has a favourable safety profile [300], it is important to carefully consider the potential risks and limitations of the procedure.

Although non-viral vectors have been explored for subretinal administration, they have generally been associated with toxicity and lower transduction levels compared to viral vectors [301]. Additionally, the viscosity of these formulations may limit their ability to adequately cover the subretinal area [302]. This highlights the importance of carefully considering the choice of vector for subretinal gene therapy.

### 9.4. Suprachoroidal Therapies

The suprachoroidal route for drug delivery, initially proposed 20 years ago, has gained increasing attention as a potential alternative to traditional subretinal and intravitreal routes [303]. This approach involves injecting a small amount of agent into the suprachoroidal space, a potential space consisting of tightly packed connective tissue between the choroid and sclera that extends from the supraciliary space at the front of the eye to the optic nerve at the back. It is bound anteriorly by the scleral spur and posteriorly by the optic nerve and posterior ciliary arteries [304]. Its structural continuity and small volume are important for understanding the potential of the suprachoroidal space as a drug delivery route, which allows for rapid dissemination of a small amount of agent around most of the eye [305]. For example, bevacizumab (149 kDa) has been shown to effectively diffuse into the choroid, RPE, and photoreceptors through this route [306]. Furthermore, the suprachoroidal route offers faster diffusion for smaller, lipophilic agents [307] and, as an ab externo approach, avoids the intraocular complications associated with intravitreal and subretinal administration [308]. Overall, the suprachoroidal route represents a promising option for delivering drugs to the outer retina.

Co-localisation with a polymer may improve particle durability and spread [309]. Nevertheless, particle clearance may be less a concern for gene vector-based therapies delivered via the suprachoroidal route. For example, suprachoroidal injection of an AAV-based vector in large animals achieved a wider distribution of outer retinal transduction than subretinal therapy [310,311,312] and lower systemic distribution and inflammation than the intravitreal route [310,312]. These experiments suggest that the suprachoroidal route is less immunogenic than the intravitreal space but more immunogenic than the subretinal space. It is possible that the exposure of these agents to the resident macrophages and lymphatic lacunae of the choroid may enhance their antigen presentation systemically, which could lead to subretinal immune cell infiltration and the neutralization of antibody production in nonhuman primates and rats [310,312].

Despite the preclinical concerns surrounding suprachoroidal viral gene therapy, the Phase 2 study of RGX-314 (developed by *REGENXBIO*, Rockville, MD, USA, in partnership with *AbbVie*, North Chicago, IL, USA; NCT04514653, https://clinicaltrials.gov/ct2/show/NCT04514653) has recently shown positive interim results (available online: https://regenxbio.gcs-web.com/news-releases/news-release-details/regenxbio-announces-additional-positive-interim-data-trials-rgx (accessed on 2 April 2023)). RGX-314 is an investigational suprachoroidal AAV-based vector that expresses an anti-VEGF antibody fragment and is being developed for the treatment of exudative AMD. These interim results indicate efficacy and limited intraocular inflammation, despite the use of a non-native protein and administration in patients who are neutralising antibody-positive without prophylactic steroid use.

Non-viral-based vectors are also currently being actively explored as alternative suprachoroidally administered therapeutics [313]. BrM may also pose a barrier to the diffusion of therapies to the retina due to its dense proteoglycan content that can sequester viral-based vectors and electrostatically repel non-viral formulations. A small molecule Factor D inhibitor (A01017, formerly developed by *Achillion Pharmaceuticals*, now by *AstraZeneca*) showed promising results in a preclinical study with biological efficacy up to three months after suprachoroidal administration in rabbits, though further studies have not been made publicly available [314]. It is unclear whether this drug was biosimilar to the orally administered danicopan currently in development.

## 10. Conclusions

The ability to visualise changes associated with age and retinal pathologies, including AMD, using high-resolution ophthalmic imaging techniques such as OCT and OCT-angiography has facilitated the study of BrM as a critical component in AMD pathogenesis. The results of the first Phase 3 trials using intravitreal complement inhibitors have brought exciting and promising advancements in the treatment of this disease that affects millions of people. Further research is needed to fully understand the transit of complement proteins and vectors across BrM and the RPE to achieve effective therapeutic delivery of inhibitors and regulators, which will be crucial in targeting complement activation that occurs on both sides of BrM.

## Figures and Tables

**Table 1:** Summary of functional and anatomical changes in BrM with age.

Functional Changes	Anatomical Changes
Decrease in elasticity Decrease in water permeability Decrease in protein permeability Decreased complement protein permeability	Accumulation of lipids, TIMP-3, MMP-2, MMP-9, calcium phosphate, and AGEs BrM thickening Reduced heparan sulphate Increased complement activation

Abbreviations: TIMP, tissue inhibitor of metalloproteinase; MMP, matrix metalloproteinase; AGEs, advanced glycation end-products; BrM, Bruch’s membrane.

**Table 2:** Changes in the composition of each layer of BrM with age.

Layers	Composition	Changes with Age
RPE basement membrane	Chondroitin sulphate Collagen IV α1–5 Collagen V Heparan sulphate Laminins 1, 5, 10, and 11 Nidogen-1	BLamD accumulation
Inner collagenous layer	Apolipoprotein E Chondroitin sulphate Clusterin Collagen I, III, and V Dermatan sulphate Fibronectin Haem Lipoproteins Vitronectin	Lipoprotein deposition BLinD accumulation
Central elastic layer	Elastin	Elastin and calcium phosphate deposition
Outer collagenous layer	Apolipoprotein E Chondroitin sulphate Clusterin Collagen I, III, and V Dermatan sulphate Fibronectin Fibulin-5 Lipoproteins	Lipoprotein deposition
Choroidal endothelial cell basement membrane	Chondroitin sulphate Collagen IV, α1, α2 Collagen V Collagen VI Endostatin Heparan sulphate Laminin	Unknown

Abbreviations: BLamD, basal laminar deposit; BLinD, basal linear deposit.

## Data Availability

Not applicable.
